# Circular RNA Signature in Lung Adenocarcinoma: A MiOncoCirc Database-Based Study and Literature Review

**DOI:** 10.3389/fonc.2020.523342

**Published:** 2020-10-09

**Authors:** Shikang Zhao, Shuo Li, Wei Liu, Yanye Wang, Xiongfei Li, Shuai Zhu, Xi Lei, Song Xu

**Affiliations:** ^1^Department of Lung Cancer Surgery, Tianjin Medical University General Hospital, Tianjin, China; ^2^Tianjin Key Laboratory of Lung Cancer Metastasis and Tumor Microenvironment, Lung Cancer Institute, Tianjin Medical University General Hospital, Tianjin, China; ^3^Department of Respiratory and Critical Care, Tianjin Medical University General Hospital, Tianjin, China; ^4^Department of Respiratory Medicine, Second Affiliated Hospital of Tianjin University of Traditional Chinese Medicine, Tianjin, China

**Keywords:** circular RNAs, lung adenocarcinoma, biomarker, sponge, MiOncoCirc

## Abstract

Circular RNAs (circRNAs) are a class of endogenous non-coding RNAs (ncRNAs) with a structure of covalently closed continuous loops, which can regulate gene expression by acting as a microRNA sponge or through other mechanisms. Recent studies have identified that the expression of candidate circRNAs are dysregulated in various tumors and hence are considered as promising diagnostic or therapeutic targets across cancer types. However, the expression and function of circRNAs in lung adenocarcinoma (LUAD) remains unclear. In this article, we investigated the expression of circRNAs in LUAD via MiOncoCirc, which is the first and comprehensive database characterizing circRNAs across >2,000 cancer samples using an exome capture RNA sequencing. We identified seven abnormally expressed circRNAs in LUAD, including circCDR1-AS, circHIPK3, circFNDC3B, circPCMTD1, circRHOBTB3, circFAM13B, and circMAN1A2, as well as conducted a literature review about the function and features of these circRNAs. Previous studies have demonstrated that circCDR1-AS, circMAN1A2, and circHIPK3 were upregulated and significantly correlated with a poor survival, or promoted the tumor progression in lung cancer, whereas other circRNAs have not been fully explored. Besides, we reviewed all the publications regarding circRNAs and LUAD, and noticed that the dysregulation of these circRNAs impacts the development of LUAD through a variety of regulatory mechanisms. In conclusion, the underlying mechanisms of aberrant expression and functions of circRNAs in LUAD are worthy of being further investigated.

## Introduction

Lung cancer, as the most common cancer, is the leading cause of cancer worldwide. Despite the improved prognosis of lung cancer patients resulting from surgery, chemotherapy, radiotherapy, tyrosine kinase inhibitor therapy, and immunotherapy, the 5-year survival rate is still low (1). Early detection with the use of low-dose CT decreases mortality in lung cancer (2). However, it is still necessary to develop more effective non-invasive biomarkers for the detection of early-stage lung cancers. As one of the main types of lung cancer, lung adenocarcinoma (LUAD) rates continue to increase in Eastern and Western populations, whereas the number of lung squamous cell carcinomas (LUSQ) gradually decreases (3). Because physicians always have to clarify diagnosis by tissue biopsy, a convenient, accurate, and non-invasive biomarker that has the ability to differentiate subtypes, and predict and monitor the progression of lung cancer is also significant.

Circular RNAs (circRNAs), first introduced by Sanger et al. in 1976 (4), are a kind of covalently closed circular non-coding RNAs (ncRNAs) without 5′ to 3′ polarity or polyadenylated tail (5). With the development of high-throughput RNA sequencing and bioinformatics tools, more and more novel circRNAs have been identified. After being reported in atherosclerotic vascular disease risk, neurological disorders, and prion diseases (6), circRNAs are also detected to play important roles in the progression of cancer (7). Owing to their unique structure, circRNAs are difficult to degrade and could be used as promising molecular biomarkers. Previous studies have confirmed that circRNAs have the potential to be diagnostic, prognostic, and predictive biomarkers (8–11). However, the biofunctions of circRNAs are still obscure. One of the major functions of circRNAs is regards miRNA sponges (12). For example, circ-AKT3 was reported to serve as a novel therapeutic to target clear cell renal cell carcinoma metastasis (13). CircFOXP1 was identified to predict and regulate the progression of gallbladder cancer (14). CircPLEKHM3 acted as a tumor suppressor in ovarian cancer (15). All these circRNAs play their role in the circRNA–miRNA–mRNA axis, although further studies still are needed to explore the function of circRNAs in lung cancer.

**FIGURE 1:**
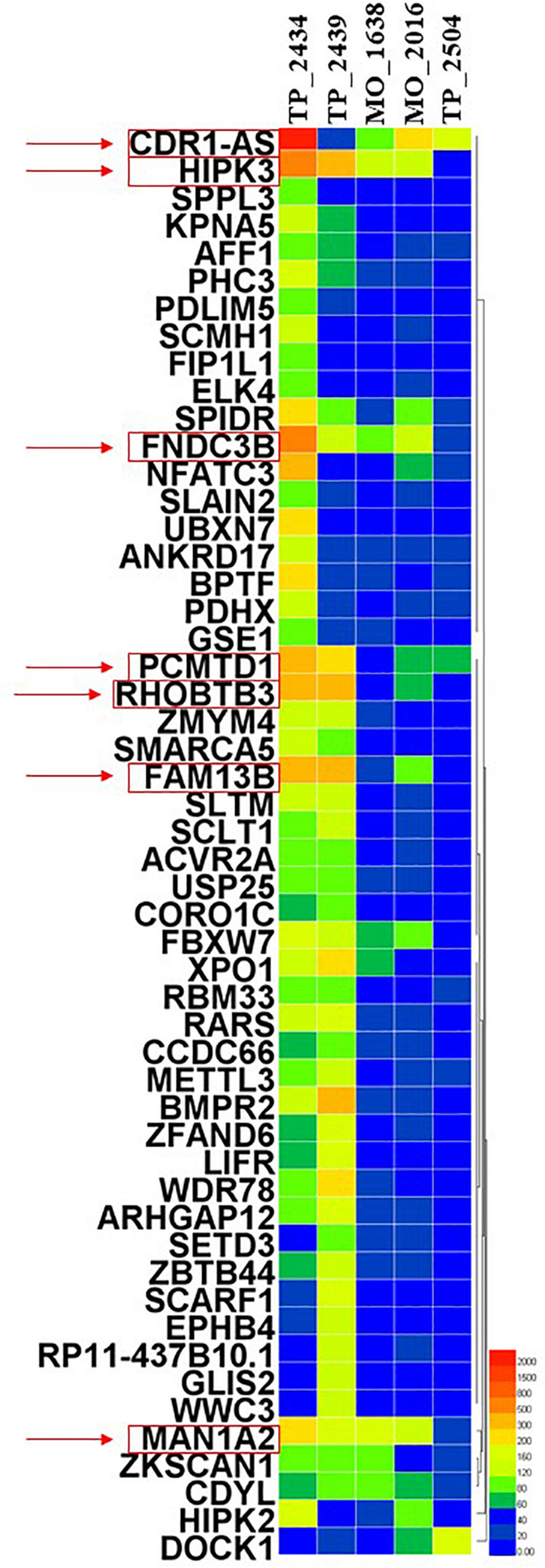
Heatmap showing circRNAs enriched in LUAD patients.

In this review, we aim to investigate the potential roles of these circRNAs in LUAD. By detecting more than 2,000 tumor samples and cell lines by exome capture RNA sequencing, researchers have collected all the data in MiOncoCirc database (16). Five patients with LUAD were selected in this database, including three female and two male patients. However, the detailed clinical information (e.g., staging and survival) for these patients was not provided in the database. To ensure the specificity of the selected circRNAs, the differentially expressed circRNAs were identified to be significant when fold-change (log2) was above 2. Relative expression heatmap based on expression of 52 different circRNAs revealed that seven candidate circRNAs have an abnormal expression profile (log2) that was above 6 (Figure 1). The detailed information of these circRNAs are summarized by searching the PubMed database. Baseline characteristics of these seven circRNAs are listed in Table 1. We further reviewed all published articles on circRNAs and LUAD and summarized their regulatory mechanisms (Table 2).

**TABLE 1:** Characteristics of circRNAs selected from MiOncoCirc database.

CircRNA	Cancer type	Source	Method	Function	Expression	Mechanism	References
circCDR1-AS	NSCLC	Tumor tissue; Cell line	qRT-PCR	Promotion(+); metastasis(+); apoptosis(−)	Up	sponge to miR-7	(17)
circHIPK3	NSCLC	Tumor tissue; Cell line	qRT-PCR	Autophagy(+); proliferation(+); migration(+); invasion(+)	Up	sponge to miR124-3p	(18)
circHIPK3	Lung cancer	Tumor tissue; Cell line	qRT-PCR	Proliferation(+) progression(+)	Up	sponge to miR-124	(19)
circMAN1A2	Lung cancer	Serum	qRT-PCR	NO	Up	sponge to miR-135a-3p*	(20)
circFNDC3B	Bladder cancer	Tumor tissue; Cell line	qRT-PCR	Proliferation(+); migration(+); invasion(+)	Down	sponge to miR-1178-3p	(21)
circPCMTD1	Glioma	Cell line	qRT-PCR	Viability(+); proliferation(+); Migration(+); invasion(+)	Up	sponge to miR-224-5p	(22)
circRHOBTB3	Clear cell renal cell carcinoma	Tumor Tissue	Microarray	NO	Down	NO	(23)
circFAM13B	Epithelial ovarian cancer	Tumor Tissue	qRT-PCR	NO	Up	NO	(24)

NO: no relevant reports; qRT-PCR: quantitative real-time polymerase chain reaction; and *not confirmed by experiment.

**TABLE 2:** Summary of circRNAs in LUAD from literature review.

Name	Cancer Type	Relate to TNM stage	Prognostic prediction	Source	Method	Function	Expression	Mechanism	References
circ-ZNF609	LAUD	NO	NO	Tumor tissue; Cell line	qRT-PCR	Proliferation(+)	Up	sponge to miR-1224-3p	(25)
circ_102231	LAUD	Associated with T and N stage	Y	Tumor tissue	qRT-PCR	Proliferation(+); invasion(+)	Up	NO	(26)
circ_0013958	LAUD	Associated with TNM stage	NO	Tumor tissue	Microarray	Proliferation(+); invasion(+); appoptosis(−)	Up	sponge to miR-134	(27)
circ_Cmras	LAUD	NO	NO	Tumor tissue; Cell line	qRT-PCR	Proliferation(+); invasion(+); migration(+); appoptosis(−)	Down	Regulate ABHD5/ATGL axis	(28)
circ_Cmras	LAUD	Associated with T stage	NO	Tumor tissue	qRT-PCR	Proliferation(+); invasion(+); migration(+)	Down	sponge to miR-567	(29)
circ-ENO1	LAUD	NO	NO	Tumor tissue; Cell line	qRT-PCR	Proliferation(+); appoptosis(−)	Up	sponge to miR-22-3p	(30)
circpvt1	LAUD	Associated with N stage	Y	Tumor tissue; Cell line	qRT-PCR	NO	Up	sponge to miR-145-5p	(31)
circ_404833	LUAD	Associated with early stage	NO	Tumor tissue; Cell line	Microarray; qRT-PCR	NO	Up	sponge to miR-149-5p*	(32)
circ_EPB41L2	LUAD	NO	NO	Tumor tissue; Cell line	qRT-PCR	Proliferation(+); invasion(+); migration(+)	Down	sponge to miR-211-5p	(33)
circcrim1	LUAD	NO	NO	Tumor tissue; Cell line	qRT-PCR	Invasion(+); migration(+); glycolysis(+); EMT(+)	Down	sponge to miR-125b-5p	(34)
circ-MTO1	LUAD	NO	Y	Tumor tissue; Cell line	qRT-PCR	Proliferation(+)	Down	sponge to miR-17	(35)
circ_0003998	LUAD	NO	NO	Tumor Tissue	qRT-PCR	Proliferation(+); appoptosis(−)	Up	sponge to miR-326	(36)
circ−TSPAN4	LUAD	Associated with TNM stage	Y	Tumor tissue; Cell line	qRT-PCR	Invasion(+); migration(+)	Up	sponge to miR-665	(37)
circ_0001946	LUAD	Associated with TNM stage	Y	Tumor Tissue; Cell line	Microarray	Proliferation(+); appoptosis(−)	Up	sponge to miR-135a-5p	(38)
circ_0006427	LUAD	Associated with TNM stage	Y	Tumor tissue; Cell line	qRT-PCR	Proliferation(+); invasion(+); migration(+)	Down	sponge to miR-6783e3p	(39)
circ_0002360	LUAD	NO	NO	Tumor tissue	High-throughput circRNA sequencing	NO	Up	sponge to hsa-mir-3620-5p	(40)
circ_0000326	LUAD	Associated with TNM stage	NO	Tumor tissue; Cell line	Microarray	Proliferation(+); migration(+); appoptosis(−)	Up	sponge to miR-338-3p	(41)
circasph	LUAD	Associated with T and N stage	Y	Tumor tissue; Cell line	High-throughput circRNA sequencing	Proliferation(+); invasion(+); migration(+)	Up	sponge to miR-370	(42)
circ_0025036	LUAD	Associated with TNM stage	NO	Tumor tissue; Cell line	qRT-PCR	Proliferation(+); appoptosis(−)	Up	sponge to miR-198	(43)
circ_0012673	LUAD	Associated with T stage	NO	Tumor tissue; Cell line	Microarray	Proliferation(+)	Up	sponge to miR-22	(44)
circrnf13	LUAD	Associated with TNM stage	NO	Tumor tissue; Cell line	qRT-PCR	Invasion(+); migration(+)	Down	sponge to miR-93-5p	(45)
circcrim1	LUAD	Associated with TNM stage	Y	Tumor tissue; Cell line	qRT-PCR	Invasion(+); migration(+)	Down	sponge to miR-93/miR-182	(46)
circ_002178	LUAD	Associated with early stage	NO	Tumor tissue; Cell line	Microarray; qRT-PCR	Promote PDL1/PD1 expression	Up	sponge to miR-34	(47)
circ_0001588	LUAD	Associated with TNM stage	Y	Tumor tissue; Cell line	qRT-PCR	Proliferation(+); invasion(+); migration(+)	Up	sponge to miR-524-3p	(48)
circprkci	LUAD	Associated with TNM stage	Y	Tumor tissue; Cell line	qRT-PCR	Proliferation(+); migration(+)	Up	sponge to miR-545/miR-589	(49)
circcdr1-AS	LUAD	Associated with T stage	Y	Tumor tissue; Cell line	qRT-PCR	NO	Up	NO	(50)
circ_0000190	LUAD	Associated with T stage	Y	Tumor tissue; Cell line	High-throughput circRNA sequencing	NO	Up	NO	(51)
circ_0005962	LUAD	No association	Y	Tumor tissue; Cell line	qRT-PCR	NO	Up	NO	(52)
circ_0003958	LUAD	No association	Y	Tumor tissue; Cell line	qRT-PCR	NO	Up	NO	
circabcc4	LUAD	NO	NO	Tumor tissue; Cell line	qRT-PCR	Proliferation(+); migration(+); appoptosis(−)	Up	sponge to miR-3186-3p	(53)
circ_0000729	LUAD	Associated with T and M stage	Y	Tumor tissue	qRT-PCR	NO	Up	sponge to miR-375	(54)
circfoxp1	LUAD	NO	NO	Tumor tissue	qRT-PCR	Proliferation(+); appoptosis(−)	Up	sponge to miR-185-5p	(55)
circ_0056616	LUAD	Associated with TNM stage	NO	Tumor tissue; Cell line	qRT-PCR	NO	Up	NO	(56)
circ-SOX4	LUAD	NO	NO	Tumor tissue; Cell line	qRT-PCR	Proliferation(+); invasion(+); migration(+)	Up	sponge to miR-1270	(57)
circ-0000211	LUAD	No association	NO	Tumor tissue; Cell line	qRT-PCR	Invasion(+); migration(+)	Up	sponge to hsa-miR-622	(58)
circ-CAMK2A	LUAD	Associated with TNM stage	Y	Tumor tissue; Cell line	qRT-PCR	Invasion(+); migration(+)	Up	sponge to miR-615-5p	(59)
circpum1	LUAD	NO	NO	Tumor tissue; Cell line	qRT-PCR	Proliferation(+); invasion(+); migration(+)	Up	sponge to miR-326	(60)

NO, no relevant reports; Y, have the ability; qRT-PCR, quantitative real-time polymerase chain reaction; and *not confirmed by experiment.

### CircCDR1as

Zhang and colleagues have found that circCDR1as exhibited a much higher expression in non-small cell lung cancer (NSCLC) compared with normal lung tissue (17). The clinicopathological characteristics of NSCLC patients revealed that circCDR1as was positively correlated with the advanced stage and poor outcome of NSCLC patients. It was also found that circCDR1as exhibited a higher expression in LUAD patients compared with lung squamous cell carcinoma (sqCC; 79.2% vs. 61.1%). Moreover, *in vitro* gain and loss of function studies using A549 and H460 cell lines identified that circCDR1as acted as a powerful miR-7 sponge/inhibitor, which could regulate epidermal growth factor receptor (EGFR), cyclin E1 (CCNE1), and phosphatidylinositol 4,5-bisphosphate 3-kinase catalytic subunit delta isoform (PIK3CD). Knockdown of circCDR1as or overexpression of miR-7 could suppress both LUAD and lung sqCC tumor cell proliferation via inducing apoptosis and G1/S arrest of NSCLC cells.

### CircHIPK3

A previous study has demonstrated that the expression of circHIPK3 was much higher in several lung cancer cell lines, which is associated with proliferation, migration, and invasion of cancer cells. In serine/threonine kinase 11 (STK11) mutant lung cancer, circHIPK3 could modulate autography through miR-124-3p-STAT3-PRKAA/AMPKα signaling. Antagonistic regulation on autophagy has also been demonstrated between circHIPK3 and linear HIPK3. Moreover, it was found that the ratio between circHIPK3 and linHIPK3, detected by RT-PCR, was correlated with poor survival, especially in advanced-stage (II, III, and IV) LUAD patients, whereas circHIPK3 or linHIPK3 alone as a biomarker is meaningless. All these results suggest that circHIPK3 is a key autophagy regulator and could be used as a potential target in STK11 mutant lung cancer (18). In addition, another study also showed that the expression of circHIPK3 was upregulated in A549 cell line and in human lung cancer tissue. Further studies indicate that overexpression of circHIPK3 is associated with high proliferation and long survival of lung cancer cells. Suppressing the expression of circHIPK3 could inhibit the survival and proliferation of lung cancer cells, but lead to a remarkable cell apoptosis. Researchers also found that circHIPK3 could act as miR-124 sponges and regulate sphingosine kinase 1 (SphK1), cyclin-dependent kinase 4 (CDK4) as well as signal transducer and activator of transcription 3 (STAT3), all of which are the targets of miR-124. In conclusion, circHIPK3 may promote the growth of lung cancer cells by sponging miR-124, and STAT3 is generally accepted as a target of miR-124 (19).

### CircMAN1A2

By comparing the serum from healthy donors and lung cancer patients, Fan et al. have found that lung cancer patients show a high level of circMAN1A2, which indicated that circMAN1A2 might be used as a potentially diagnostic biomarker in lung cancer patients. Bioinformatic analysis suggested that circMAN1A2 was most likely combined with has-miR-135a-3p, which was previously reported to be downregulated in ovarian cancer and inhibit cancer cell growth. Thus, circMAN1A2 seemed to exert its biofunction by acting as miR-135a-3p sponges. Further bioinformatic analysis indicated that solute carrier family 4 member 8 (SLC4A8), IKAROS family zinc finger 4 (IKZF4), small cell adhesion glycoprotein (SMAGP), Sp1 transcription factor (SP1), erb-b2 receptor tyrosine kinase 3 (ERBB3), and chromobox 5 (CBX5) were mostly probable binding partners for hsa-miR-135a-3p (20). However, there is no histology-based subgroup analysis in this study, and hence further research is necessary to explore its regulatory mechanism in LUAD and lung sqCC, respectively.

### Other CircRNAs

The expression and function of circRNAs, circFNDC3B, circPCMTD1, circRHOBTB3, and circFAM13B, which express high in LUAD in our database analysis, have not been reported in lung cancer in previous studies. However, some of these circRNAs expressed with an abnormal level and participated in oncogenesis in other cancers. It was demonstrated that the expression of circFNDC3B was downregulated in bladder cancer tissue and cell lines, and interfered on tumor growth via miR-1178-3p/G3BP2/SRC/FAK axis (21). In glioma patients, circPCMTD1, as a newly discovered circRNA, was able to promote the progression of glioma by sponging to miR-224-5p (22). By using microarray analysis and statistical methods, Franz et al. have demonstrated that circRHOBTB3 could act as a diagnostic and prognostic biomarker of clear cell renal cell carcinoma (23). Besides, it was reported that circFAM13B was decreased in the epithelial ovarian cancer specimens, whereas the specific regulatory mechanism remained unclear (24).

To more comprehensively explore the detailed function of circRNAs in LUAD, we reviewed all the publications regarding circRNAs and LUAD (Table 2). The most common regulatory mechanism of these circRNAs is acting as miRNA sponges, and one circRNA could sponge with more than one miRNA which impacts the tumor progression. Some studies also suggested that circRNAs could bind protein to regulate LUAD cell progression. In these studies, the abnormally expressed circRNAs were correlated with LUAD cell proliferation, invasion, migration, apoptosis, epithelial–mesenchymal transition, and drug resistance. Some of these circRNAs were also related to the TMN stage as well as prognosis in LUAD patients (25–60). Moreover, a previous study indicated that circRNA-002178 could enhance PD-L1 expression by absorbing miR-28-5p in CD8^+^ T cells (47).

## Discussion

Circular RNAs, a large class of ncRNAs, have been demonstrated to dysregulate in LUAD patients. From the current studies, the abnormal expression of circRNAs is correlated with the poor prognosis, disease progression, metastasis, and recurrence. Most of these circRNAs are isolated from tissues and tumor cells. There are some reports suggesting that the abnormal expression of circRNAs could be found in plasma from cancer patients (61–63). Furthermore, Wang et al. demonstrated that circRNAs had the potential role to distinguish LUAD and LUSQ, indicating that circRNAs may play a different role in the initiation and development of LUAD and LUSQ (64). In recent years, owing to the popularization of CT as a diagnostic method of early lung cancer, the mortality rate of lung cancer patients has been significantly reduced (65). However, it is not easy to distinguish benign and malignant nodules in many cases, and it is also difficult to differentiate the types of lung cancers. Previous studies have reported that this special class of endogenous ncRNAs could be considered as reliable biomarkers for the diagnosis and evaluation in cancers because of their stable structure and convenient way to obtain.

It is well known that circRNAs exert their biofunctions by playing the role of miRNA sponges. In this review, we have noticed that most circRNAs work in this way. For example, circCDR1 maintains the expression of EGFR, CCNE1, and PIK3CD by acting as a sponge of miR-7. Besides, there are other reports suggesting that circRNAs are able to exert their biofunctions by other approaches, such as acting as sponges or decoys for proteins, directly interacting with specific proteins to enhance their function, functioning as protein scaffolds to promote their reaction kinetics, recruiting proteins, and combining with ribosome to play the role of templates for translation (66). However, most of these studies investigated more about the dysregulated circRNAs in tumor tissues or cell lines instead of peripheral blood. Thus, further studies are needed to explore the detailed functions of circRNAs in different resources.

Although targeted therapy and immunotherapy are widely used in LUAD, immunotherapy is only efficient in approximately 20% of all patients and targeted therapy could only benefit those patients with specific gene mutations. Also, all the patients would develop drug resistance inevitably. Thus, it is urgent to find novel treatment strategies. Considering the essential role of circRNAs, whether these endogenous ncRNA could be the potentially therapeutic target deserves further investigation. Moreover, owing to the difficulty of tissue biopsy in many cancer patients, plasma circRNAs may be a promising non-invasive biomarker in the diagnosis, individualized treatment evaluation, and dynamic follow-up. From the literature review (Table 2), we found that most of the listed circRNAs were correlated with the TNM stage and prognosis in LUAD patients, suggesting that a panel of circRNAs may have clinical value in the diagnosis and predicting prognosis in LUAD patients. However, the exact mechanisms of some circRNAs in LUAD are still not clear, which need further investigation.

In conclusion, circRNAs could be considered as not only important biomarkers for early diagnosis and prognosis of LUAD but also the potential targets for LUAD treatment.

## Data Availability Statement

The datasets analyzed for this study can be found in the MiOncoCirc database (https://mioncocirc.github.io/).

## Author Contributions

SKZ wrote the article. SL, WL, YYW, XFL, SZ, and XL completed the figures and tables. SX conceived, organized, and edited the text. All authors contributed to the article and approved the submitted version.

## Conflict of Interest

The authors declare that the research was conducted in the absence of any commercial or financial relationships that could be construed as a potential conflict of interest.
